# Similarities and Differences in the Peripheral Actions of Thyroid Hormones and Their Metabolites

**DOI:** 10.3389/fendo.2018.00394

**Published:** 2018-07-19

**Authors:** Ruy A. Louzada, Denise P. Carvalho

**Affiliations:** Laboratorio de Fisiologia Endocrina Doris Rosenthal, Instituto de Biofísica Carlos Chagas Filho, Universidade Federal do Rio de Janeiro, Rio de Janeiro, Brazil

**Keywords:** 3, 5-T2, T1AM, thyroid hormone, deiodinase, thyroid hormone analogs

## Abstract

Thyroxine (T4) and 3,5,3'-triiodothyronine (T3) are secreted by the thyroid gland, while T3 is also generated from the peripheral metabolism of T4 by iodothyronine deiodinases types I and II. Several conditions like stress, diseases, and physical exercise can promote changes in local TH metabolism, leading to different target tissue effects that depend on the presence of tissue-specific enzymatic activities. The newly discovered physiological and pharmacological actions of T4 and T3 metabolites, such as 3,5-diiodothyronine (3,5-T2), and 3-iodothyronamine (T1AM) are of great interest. A classical thyroid hormone effect is the ability of T3 to increase oxygen consumption in almost all cell types studied. Approximately 30 years ago, a seminal report has shown that 3,5-T2 increased oxygen consumption more rapidly than T3 in hepatocytes. Other studies demonstrated that exogenous 3,5-T2 administration was able to increase whole body energy expenditure in rodents and humans. In fact, 3,5-T2 treatment prevents diabetic nephropathy, hepatic steatosis induced by high fat diet, insulin resistance, and weight gain during aging in Wistar male rats. The regulation of mitochondria is likely one of the most important actions of T3 and its metabolite 3,5-T2, which was able to restore the thermogenic program of brown adipose tissue (BAT) in hypothyroid rats, just as T3 does, while T1AM administration induced rapid hypothermia. T3 increases heart rate and cardiac contractility, which are hallmark effects of hyperthyroidism involved in cardiac arrhythmia. These deleterious cardiac effects were not observed with the use of 3,5-T2 pharmacological doses, and in contrast T1AM was shown to promote a negative inotropic and chronotropic action at micromolar concentrations in isolated hearts. Furthermore, T1AM has a cardioprotective effect in a model of ischemic/reperfusion injury in isolated hearts, such as occurs with T3 administration. Despite the encouraging possible therapeutic use of TH metabolites, further studies are needed to better understand their peripheral effects, when compared to T3 itself, in order to establish their risk and benefit. On this basis, the main peripheral effects of thyroid hormones and their metabolites in tissues, such as heart, liver, skeletal muscle, and BAT are discussed herein.

## Introduction

Thyroid hormones affect development, growth, and metabolic control, therefore being indispensable to normal development and body energy expenditure (1).

Thyroid dysfunction such as hypothyroidism is implicated in growth and developmental impairment, changes of lipid, and cholesterol metabolism, cardiovascular diseases, and a decreased metabolic rate. In contrast, hyperthyroidism is a catabolic syndrome that is related to increased metabolic rate, tachycardia, and loss of lean body mass (2).

The thyroid gland produces both thyroxine (T4) and 3,5,3'-triiodothyronine (T3) (3) and every tissue expresses the enzymes that are able to metabolize these hormones, the so-called iodothyronine deiodinases that remove iodine atoms from iodothyronines. Type 1 deiodinase (D1) is found in the thyroid, liver, and kidneys, while type 2 deiodinase (D2) is expressed in the central nervous system, human thyroid, skeletal muscle, and brown adipose tissue (BAT), and both of them convert T4 into T3 through the outer ring deiodination reaction. Type 3 deiodinase (D3) inactivate T4 by its conversion into reverse T3 (rT3) through the inner ring deiodination that can also be catalyzed by D1. Under physiological conditions, D3 is expressed in the brain, placenta, and pancreas, but can be induced in other tissues under pathophysiological circumstances.

The activity of deiodinases impact both serum and tissue levels of T4 and T3, as described when animals are exposed to cold temperatures (4). Also, the regulation of decidua and placenta deiodinase activities correspond to the best example of space temporal regulation of thyroid hormones metabolism that impact on the fetus physiology (5–8). Recently, several thyroid hormone metabolites have been detected in human placenta, and future studies are necessary to address their possible effects during pregnancy (9).

It has been proposed that D1 contributes to serum T3 concentrations due to its catalytic site that is believed to face the extracellular space, while D2 is an endoplasmic reticulum resident protein that is important for intracellular T3 availability, and T3 receptor saturation in tissues. Besides the deiodinase reactions, TH undergo tissue-specific metabolism that includes sulfation, glucuronidation, deamination, and decarboxylation (1).

Since both T3 and rT3 are substrates for deiodinases, diiodothyronines are also produced, namely 3,5-diiodothyronine (3,5-T2), 3,3'-diiodothyronine (3,3'-T2), and 3',5'-diiodothyronine (3',5'-T2). Iodothyronines can also be decarboxylated giving rise to a phenethylamine derivative called a thyronamine (TAM), such 3-iodothyronamine (T1AM) (10). 3,5-T2, and T1AM are the most studied metabolites to date and they play significant physiological roles. The endogenous presence of these metabolites was confirmed by the development of specific immunoassays to detect 3,5-T2 (11) and T1AM (12).

Thyroid hormones actions can be separated into two major groups: (1) the central effects that consist of a direct signaling on the central nervous system, and (2) the peripheral effects that correspond to direct effects in responsive tissues. T3 controls energy expenditure via central and peripheral pathways. For example, T3 stimulates specific neurons of the ventromedial nucleus, which activate the sympathetic nervous system that in turn innervates the BAT and leads to adaptive thermogenesis (13); concomitantly, T3 acts directly in the BAT and activates the thermogenic program by the control of lipid metabolism and uncoupling protein 1 (UCP1) activation (14). The aim of this review is to discuss the peripherical effects of thyroid hormones and their metabolites isolating the similarities and differences in their actions and the promising use of 3,5-T2 and T1AM as therapeutic agents.

## Biosynthetic routes of TH metabolites 3,5-T2, and T1AM

In terms of structure, thyronamines differ from thyroid hormones and their deiodinated derivatives due to the absence of the carboxylate group in the beta-alanine side chain (15). Therefore, it has been suggested that T1AM is produced from TH precursors by both deiodination and decarboxylation reactions. However, the sequence of reactions for thyronamines biosynthesis and the organs where they occur are still poorly defined.

3-iodothyronamine (3-T1AM) production was believed to occur in the thyroid, to be dependent on the presence of the sodium-iodide symporter and thyroperoxidase, and did not seem to involve extrathyroidal metabolism of T4 (16). However, FRTL-5 cells incubated with T4 were unable to produce T1AM (17). *In vitro* experiments also showed significant 3-T1AM production in H9c2 rat cardiomyoblasts exposed to T3, which questions the importance of thyrocytes for thyronamine production (18).

**Figure 1:**
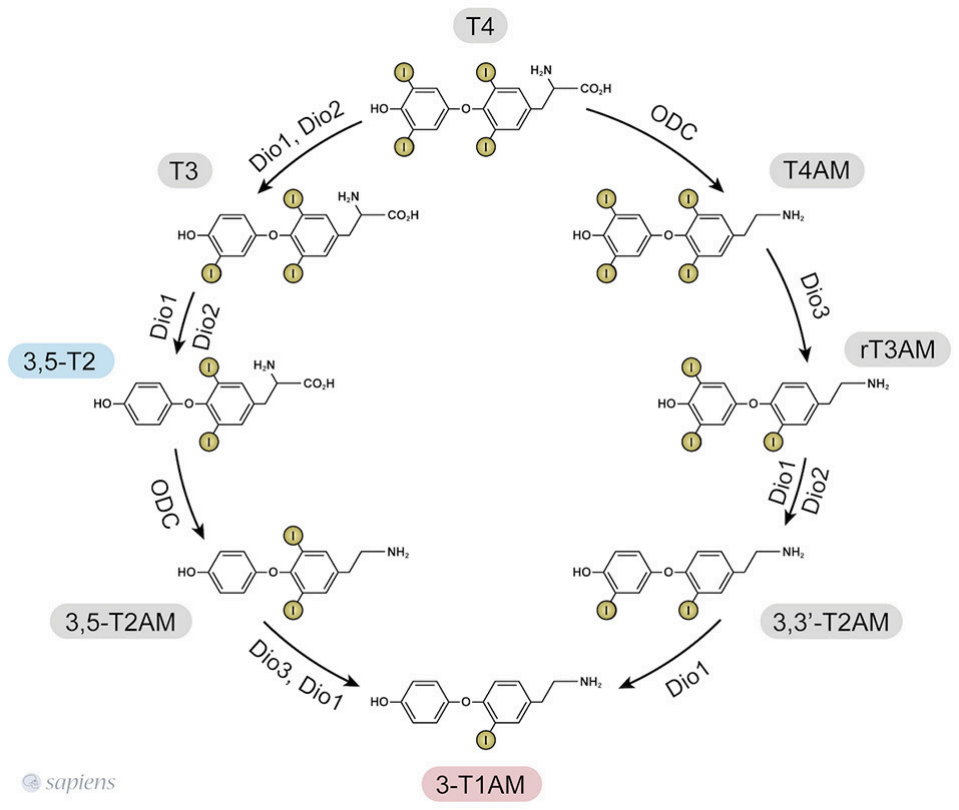
3,5-T2, and 3-T1AM biosynthetic pathways from thyroid hormones T4 and T3, as identified in murine intestinal tissue (15, 25). ODC—ornithine decarboxylase. Dio—iodothyronine deiodinase.

T1AM synthesis from TH requires the decarboxylation of the L-amino acid moiety. Initially, it was proposed that the aromatic L-amino acid decarboxylase (AADC) (19) mediated T1AM biosynthesis, but it has recently been demonstrated that patients lacking functional AADC activity exhibited normal serum 3-T1AM levels (20). The generation of extrathyroidal TH metabolites was confirmed due to the detection of 3,5-T2 in thyroidectomized individuals (11) and the finding that serum T1AM was even higher in thyroidectomized and radioiodine-treated patients than in healthy individuals (12). The studies in T4-substituted thyroid cancer patients lacking functional thyroid tissue suggest extrathyroidal 3-T1AM production, whereas studies using labeled T4 in mice indicate intrathyroidal formation. The contradictory results might be due to the different administrations routes, once thyroidectomized individuals received T4 orally, whereas the mice received it intraperitoneally (21). Elegantly, it was shown that the intestine can produce 3-T1AM from T4 and 3,5-T2 (21) due to the expression of the molecular machinery required for 3-T1AM biosynthesis: ornithine decarboxylase (ODC) and all the three deiodinase isoforms (D1, D2, and D3). Consequently, ODC was identified as the first enzyme able to decarboxylate thyroid hormones (21). Purified human ODC can, in fact, mediate decarboxylation of T4 and 3,5-T2 to T4AM, and 3,5-T2AM, respectively, as shown in Figure 1 (21).

Based on the ability of ODC to decarboxylate only T4 and 3,5-T2 (21), the only way to produce other thyronamines is from T4AM and T2AM. However, cardiac cells exposed to T3 were able to yield T1AM (18). Since purified ODC does not decarboxylate T3 to yield T3AM (21), one can speculate that T3 might be deiodinated into 3,5-T2 prior to the decarboxylation step.

Since the one step iodothyronine decarboxylation reaction to yield thyronamine was identified, the following question was posed: could thyronamines be metabolized by iodothyronine deiodinases? D1 exhibits both phenolic and tyrosyl ring deiodination activities (Figure 1), while D2 and D3 are specific for to the position of deiodination. D2 only catalyses the phenolic or outer ring deiodination, e.g., the conversion of T4 into T3, whereas D3 only catalyses the deiodination of the tyrosyl or inner ring, e.g., the conversion of T4 into rT3.

Interestingly, rT3AM can be readily deiodinated by all the three deiodinase isozymes, such as occurs with rT3 (15). However, apparently ODC does not convert rT3 into rT3AM (21), suggesting that the source of rT3AM is T4AM. Therefore, a synchronized order of events involving decarboxylation and deiodination takes place for T1AM formation. In contrast, T4AM is not a substrate for neither D1 nor D2 but is instead a substrate of D3, leading to rT3AM formation. Deiodination of rT3AM by D1 and D2 then produce T1AM, thus providing a specific biosynthetic pathway for endogenous T1AM production from T4AM, which would result from the decarboxylation of T4 followed by D3 deiodination (15, 22). The 3,5-T2, and 3-T1AM biosynthetic routes from T4 are shown in Figure 1.

## Endogenous levels of TH metabolites 3,5-T2, and T1AM

Serum levels of 3,5-T2 have been reported to be around 0.24 nM in euthyroid humans (23), and they seem not to differ in thyroid dysfunction states (11). However, a study reported reduced free T3 levels together with elevated 3,5-T2 levels in patients with postoperative atrial fibrillation (24). 3,5-T2 might also be present in tissues, but so far it has not been possible to detect endogenous 3,5-T2 in heart tissue (10), but 1.5 fmol of 3,5-T2/100 g tissue could be detected in rat liver (25).

T1AM was detected in brain within different ranges such as: 0.4 (26), <1 (27) and 49 pmol/g of tissue (28). T1AM (pmol/g of tissue) is found at different levels in different rat brain regions, such as: 60.4 in cerebral cortex, 20.9 in hemisphere white matter and 23.2 in cerebellum (18). In human serum, T1AM was detected in the range of 0.15–0.20 pmol/ml, while 0.3 pmol/ml were detected in rat serum (18). T1AM (pmol/g of tissue) was also identified in concentrations higher than the serum levels in many other rodent tissues, such as: heart (6.6), liver (92.9), kidney (36.08), skeletal muscle (25.02), stomach (15.46), and lung (5.6) (18). Other studies found 7 pmol T1AM/g (29) and 68 pmol T1AM/g (30) of rat liver, showing a variable value depending of the species and the methods of analyses used.

Recently, a study has compared the endogenous T1AM levels in different tissues and its tissue levels after 7 days of administration of two different T1AM doses (1 mg and 5mg/100 g b.w.) (31).

Endogenous T1AM and 3,5-T2 are generated from decarboxylation and deiodination reactions catalyzed by enzymes that expressed in different tissues (18, 21, 32–34). Thus, it is possible that the regulation of the local synthesis of these metabolites might also be important for their physiological effects.

## Regulation of oxygen consumption by thyroid hormone metabolites

T3 is able to increase oxygen consumption and decrease body fat, however the undesirable side effects of T3 administration limit its therapeutic use for the control of metabolic disorders, which favored the development of TH analogs designed to exert no deleterious effects on the heart and lean body mass loss (35).

Almost 30 years ago, the TH metabolite 3,5-T2 was shown to increase oxygen consumption in isolated hepatocytes (36), what was afterwards corroborated using blood mononuclear cells (37) and also *in vivo* in rats (38, 39), mice (40), and humans (41). Since the metabolic effect of 3,5-T2 was faster than T4 or T3 treatment, and without the need of new protein synthesis, the authors postulated that 3,5-T2 could act trough a post-translational mechanism, independently of genomic action [For recent review see (42)].

Both T3 and 3,5-T2 were able to increase the basal metabolic rate to the same extent in hypothyroid rats induced by propylthiouracil (PTU) and iopanoic acid administration (43). However, a single dose of 3,5-T2 was not sufficient to increase the metabolic rate in euthyroid rats differently from T3, but the chronic treatment with 3,5-T2 increased metabolic rate in aging euthyroid rats (39) and in high fat diet fed euthyroid rats (38). In contrast, a single dose of T1AM rapidly induced a hypometabolic state in rodents and hamster, with a significant decrease in the core temperature during the first hours after administration (27, 44) and persisting for 8 days of administration in mice (45). This opposite action show that thyroid hormones metabolites might also counteract the classical actions mediated by T3. However, even though T1AM has opposite effects from those of 3,5-T2 on oxygen consumption, both stimulate lipolysis, as produced by T1AM administration to spontaneously overweight mice (45) and by 3,5-T2 treatment of rats fed a high fat diet (HFD) (38).

The metabolic pathways implicated in these effects described above will be discussed in the next section separately, isolating each peripherical tissue that can contribute to the higher oxygen consumption rate, such as skeletal muscle, BAT, and liver.

T3 positively regulates mitochondria function and induces mitochondrial biogenesis. The peroxisome proliferator activated receptor gamma (PPAR gamma) co-activator 1α (PGC-1α) is a transcriptional co-activator regulated by T3 that mediates biological programs related to energy metabolism that coordinates mitochondrial biogenesis (46). PGC-1α cDNA was first cloned from BAT, and the protein was described to be involved in the induction of UCP1 (47). To date, it has been accepted that PGC-1α plays indispensable roles in glucose and fatty acid metabolism, mitochondrial biogenesis and adaptive thermogenesis (48). Notably, the PGC-1α gene contains a TH responsive element (49) and both T3 (50) and 3,5-T2 (51) rapidly induce PGC-1α expression.

## Genomic and non-genomic actions of thyroid hormones

**Figure 2:**
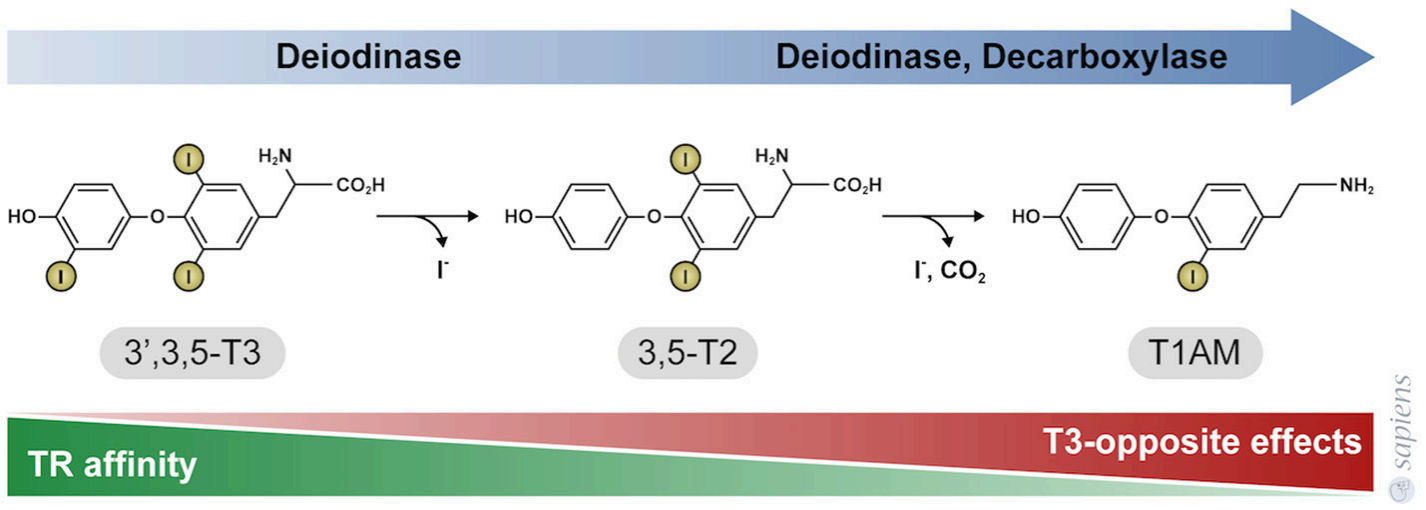
Thyroid hormone metabolites 3,5-T2, and 3-T1AM. 3,5-T2 is generated by the outer ring deiodination of T3, and T1AM is generated by enzymatic deiodination and decarboxylation of T4 or 3,5-T2, as shown in Figure 1. The affinity of thyroid hormone nuclear receptors (TR) for these metabolites is significantly lower, when compared to T3. TR affinity is approximately 500-fold lower for 3,5-T2, whereas TR do not bind to T1AM that in turn exerts effects that are opposite to T3 and 3,5-T2 actions.

T3 primarily exerts its effects by binding to thyroid hormone nuclear receptors (TR) that affect gene transcription through thyroid hormone responsive elements (TRE) in the promoter region of target genes. TR alpha and beta isoforms are encoded by two genes that are differentially expressed in various tissues. The distribution of these receptors is heterogeneous among the different tissues, and as a result some physiological effects of T3 are TR isoform specific. For example: TR beta plays an essential role in the negative regulation of thyroid stimulating hormone (TSH) secretion (52), whereas TR alpha mediates the positive chronotropic effects of thyroid hormones in the heart (53). Moreover, some physiological effects might require the activation of both TR alpha and TR beta in the same tissue, such as occurs in BAT. Although previous studies claim that TH metabolite 3,5-T2 might only act through non-genomic mechanisms, recent studies have demonstrated that both 3,3-T2, and 3,5-T2 can weakly interact with both TR alpha and TR beta, when compared with T3 (Figure 2) (54, 55). Despite the lower affinity of 3,5-T2 for TR beta (55), its ability to exert negative feedback at the hypothalamus-pituitary axis (39) indicate that 3,5-T2 pharmacological actions might also be dependent on the *in vivo* TR beta transactivation.

3,5-T2, and T3 interact with long and short TR beta1 isoforms in teleost, respectively (55, 56). These findings strongly suggest the existence of different signaling pathways for hormone and its metabolite, indicating that the specific response can be, at least in part, due to the differential expression of these receptors. T3 upregulates TR beta, whereas 3,5-T2 downregulates the long TR beta1 isoform (57).

Thyroid hormones can also act through non-genomic mechanisms by binding to sites in the plasma membrane, such as alpha V beta 3 integrin (58), and the activation of cytoplasmic proteins such as AMPK, PI3K/Akt (59) and MAPK (60). Similarly, 3,5-T2 is as a potent stimulator of the same signaling pathways involving AMPK (38) and PI3K/Akt (59), showing a similar partner of non-genomic actions when compared to T3.

Another cellular target of 3,5-T2 is the mitochondria. 3,5-T2 may stimulate cellular respiration via a direct action involving mitochondria by the interaction with cytochrome C Oxidase (COX) to impair the allosteric ATP inhibition of COX, which decreases the respiratory efficiency (61). 3,5-T2 also increases Sirtuin 1 activity, a nuclear deacetylase (62). Interestingly, T3 is not able to stimulate COX (63) and Sirtuin 1 (62) to the same extent as 3,5-T2, showing important differences concerning the non-genomic actions of these hormones.

T1AM has no affinity for TR beta or TR alpha [Figure 2; (30)]; T1AM can act through different mechanisms depending of the cellular type (64, 65), such as via the trace amine-associated receptor that activate the adenylyl cyclase and protein kinase A signaling pathway (66), the inverse agonist action on the alpha 2A -adrenergic receptor (67), the stimulation of the transient melastatin 8 channel receptors, increasing intracellular calcium and MAPK ERK1/2 pathway (68, 69) and the Sirtuin 6 and 4 function (31, 70). Additionally, T1AM also inhibits mitochondria F0F1 ATPase activity (71).

## Thyroid hormones, their metabolites and the hypothalamus-pituitary-thyroid axis

Thyroid gland is under the positive control of TSH, and in turn T4 and T3 negatively control the hypothalamus-pituitary axis, through a classical negative feedback loop. T3 downregulates the secretion of Thyrotropin Releasing Hormone (TRH) in the medio basal hypothalamus and of TSH in the pituitary. Locally produced T3 originates from T4 deiodination by D2 (72), and the metabolite 3,5-T2 is also able to downregulate hypothalamic TRH mRNA expression and serum TSH levels in rats (39), leading to central hypothyroidism, what was also confirmed in mice (40, 54). However, the only study in 2 human volunteers demonstrated that 3,5-T2 for 3 weeks did not change serum thyroid hormone levels despite the stimulating effect on metabolic rate (41).

The intracerebroventricular administration of T1AM decreased plasma free T3 in mice (73), and more recently, 3-T1AM was shown to act directly on the thyroid gland (74), since the administration of 3-T1AM to mice for seven days decreased the thyroid mRNA contents of NIS, thyroglobulin, and pendrin but did not interfere with the hypothalamus-pituitary axis (74).

## Thyroid hormone metabolites and the heart

Thyroid hormones are required to maintain heart rate, myocardial contractility and vascular function (75). The genomic effects of TH on the heart that control chronotropic and inotropic features are mainly mediated by the TH-specific receptor TR alpha1 (53). High T3 levels are related to increased myocardial contractility and electrical conduction, and arrhythmias events, while low T3 promotes opposite effects, such as bradycardia (10). As a result, both hyper-and hypothyroidism are important risk factors for cardiovascular diseases.

The heart is able to locally adjust the metabolism of TH through the modulation of D3 activity, as described in myocardial infarction (76) and dilated cardiomyopathy (77). Thus, ischemic insults promote local hypothyroidism, which results in the lower expression of T3 responsive genes that are involved in contractile apparatus and energy metabolism, what is interpreted as a counter regulatory mechanism to reduce oxygen consumption after an ischemic event (78). Animals deficient of D3 show a worst infarct progression (77), showing the importance of cardiac D3 induction to attenuate the classical T3 effects and/or produce other metabolites that could play a key role in disease prognosis.

Critically ill patients usually present decreased circulating T3 levels, what is called the “low T3 syndrome” or the “euthyroid sick syndrome” (79). The low serum T3 is at least in part a consequence of the higher D3 activity that is induced in some tissues (76, 78). The relevance of thyroid hormones to cardiac function motivated several authors to test thyroid hormones replacement after myocardial infarct (MI). Many strategies were adopted including treatment some hours after MI (at 72h after MI) (80) or 13 weeks after MI (81). In summary, T3 effects on cardiac remodeling is listed: (1) increased mitochondrial content due higher PGC-1α expression after T3 treatment (82), (2) decreased fibrosis development due to increased metalloproteinases expression (83), )3) decreased apoptosis signaling from mitochondrial (82, 84), and (4) activation of ERK to promote angiogenesis in myocardium (85, 86). Most of these effects were considered to be secondary to a positive effect on mitochondria, which is a classical target of T3 (80, 87). To our knowledge, the 3,5-T2 treatment after MI has not been assessed so far. Some encouraging results showed that 3,5-T2 treatment was potentially able to modulate the same pathways as T3 in skeletal muscle (59), in BAT (51), in liver (62) and in kidney (88), indicating that 3,5-T2 might also be a very promising agent in cardiac diseases.

Chronic 3,5-T2 treatment did not significantly change neither heart weight (38, 39) nor heart rate (39, 89). However, higher doses of 3,5-T2 caused cardiac hypertrophy in rats and mice, just as T3 does (39, 40, 54). In humans, the only clinical study using low doses of 3,5-T2 in two volunteers showed a significant increase in resting metabolic rate, without changes in cardiac function evaluated by echocardiography (41).

It has been demonstrated that the prejudicial cardiac remodeling is mediated in part by overactivation of sympathetic nervous system from the paraventricular nucleus PVN (90). To isolate the central from the direct peripherical cardiac effects of thyroid hormones, some studies have tested the ischemic and reperfusion model using isolated hearts. (91) demonstrated a protection against reperfusion injury mediated by TR alpha, when acute T3 administration preceded the ischemic insult (91), however the possible cardioprotective effects of 3,5-T2 have not been reported yet. Recently, 3,5-T2 increased glucose consumption in isolated rat hearts, contrary to what was observed for T3 and T4 in the same conditions. This direct 3,5-T2 effect on cardiomyocytes was not associated with alterations in contractile performance (92).

**Figure 3:**
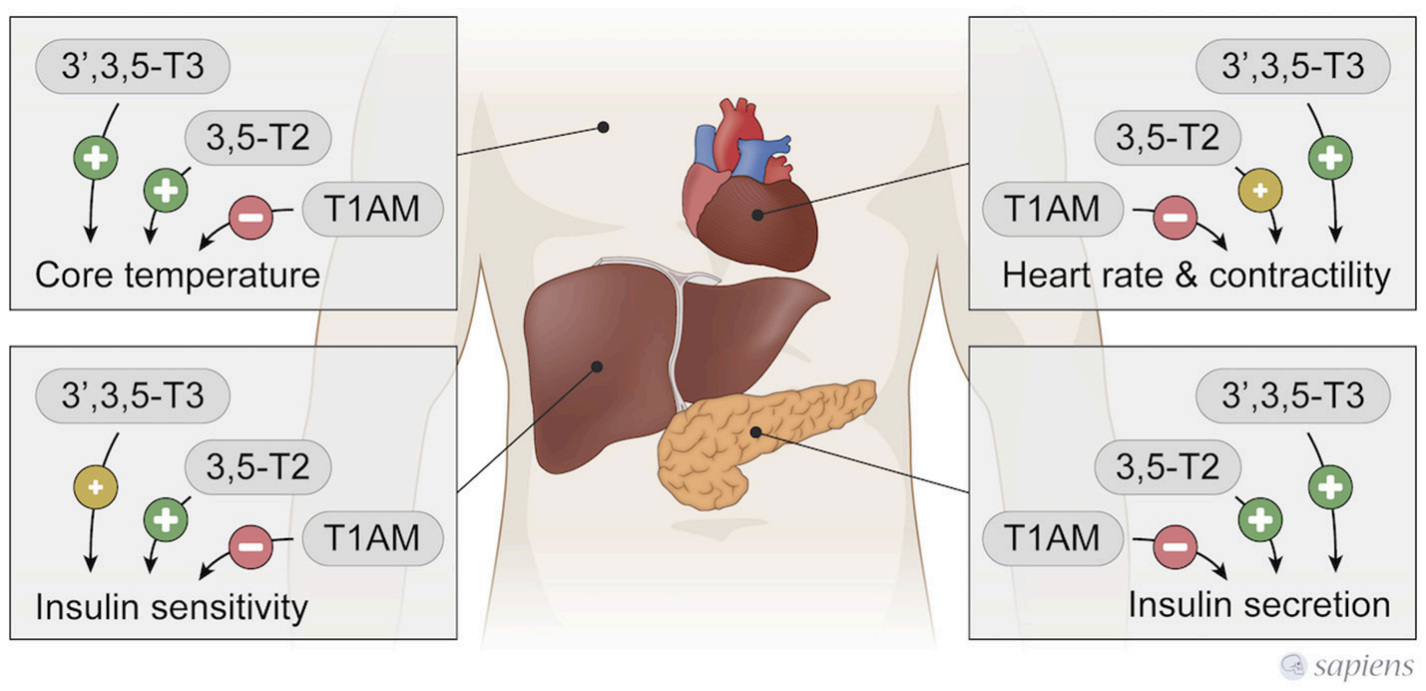
T3, 3,5-T2, and T1AM actions in peripherical tissues. 3,5-T2, and T3 have similar actions, while T1AM exerts T3 and 3,5-T2 opposite effects.

In contrast, T1AM treatment in mice (27) or administered to isolated rat hearts (30, 93) induced bradycardia, which is opposite to the classical T3 effect (Figure 3). Furthermore, T1AM protected hearts against ischemia without significant hemodynamic actions (94). The balance between D2 and D3 activities could favor the formation of different metabolites in the cardiomyocyte, but further studies are necessary for the comprehension of how the thyroid hormones are metabolized in the heart under different pathophysiological conditions, and whether these different metabolites may play a key role in the regenerative process and cardiac remodeling.

## Thyroid hormones metabolites and the brown adipose tissue

The overall cellular effects of T3 that impact on energy expenditure are based on its ability to decrease the efficiency of ATP synthesis and increase its turnover. T3 stimulates thermogenesis and thus allows the maintenance of body internal temperature under cold exposure (2, 95). Facultative thermogenesis takes place mainly in the BAT and it involves several mechanisms, such as the disruption of the proton gradient across the inner mitochondrial membrane, which mediated by the mitochondrial UCP1 (96). In mitochondria, the energy derived from the electrochemical proton gradient across the inner mitochondrial membrane is used by ATP synthase to produce ATP. The passive flux of protons (proton leak) through the inner membrane through UCP1, dissociates oxygen consumption in the electron transport chain from ATP synthesis, and thus the energy is somehow dissipated and energy expenditure increases (95–97).

Facultative thermogenesis is T3 dependent, as illustrated by the fact that hypothyroid rats do not survive after few hours of cold exposure (98). During cold exposure, an intense activity of sympathetic flux and norepinephrine (NE)/cAMP/protein kinase A signaling activates D2 activity in BAT (99) to increase the local T3 production and consequently thyroid hormone receptor saturation (4). Local T3 controls the expression of many proteins implicated in the turnover of lipid metabolism (14), and lipogenesis is very important for the maintenance of lipid stores that will be used to provide free fatty acids to activate UCP1. Like hypothyroid mice, the D2 deficient mice are unable to survive in low temperature for a long period of time. Apart from the mitochondria effects, T3 also increases the cell membrane permeability to Na^+^ and Ca^+^ ions and activate the Na^+^/K^+^ ATPase pump, increasing heat dissipation due to ATP hydrolysis. Among all the cell adaptations discussed before, mitochondria biogenesis is a key event for thermogenesis process (47).

3,5-T2 administration restores the thermogenic program of BAT from hypothyroid rats mainly through mitochondria activation, what is similar to T3 effects (51, 100). To investigate the possible central effect of 3,5-T2 in the cerebral nuclei that control neuronal sympathetic activation, the authors demonstrated that the turnover of NE in BAT was not increased in hypothyroid rats treated with 3,5-T2. In turn, hypothyroid rats treated with either T3 or 3,5-T2 showed the same induction levels of PGC-1α, showing a direct peripheral effect of 3,5-T2. On the other hand, T3 is also able to increase the sympathetic flow to BAT through hypothalamic AMPK inhibition (13), while 3,5-T2 does not show this central effect. The mechanism of action of 3,5-T2 in BAT is not completely defined, although both TR alpha and TR beta seem to mediate the regulatory effects of T3 on facultative thermogenesis (101). It has been proposed that TR alpha induces lipolysis in synergism with the adrenergic system, while the induction of UCP1 expression is mediated by TR beta (101).

In contrast to 3,5-T2 (51), the TR beta agonist (CG-1) did not restore the normal thermogenic function in hypothyroid mice. BAT UCP1 levels are normal in hypothyroid mice treated with GC-1, although they remain cold intolerant due to inadequate BAT cellular response to norepinephrine (101).

In contrast to 3,5-T2 and T3 thermogenic effects, a single T1AM administration rapidly induces a hypometabolic state in rodents, acutely decreasing body temperature in a dose dependent manner (27, 44). In fact, T1AM induces profound hypothermia, however the mechanisms involved are not completely known (Figure 3). T1AM administration induce tail vasodilatation, which could lead to heat loss; however, this effect seems to occur through central mechanisms, since the intracerebroventricular administration of T1AM also produces vasodilatation secondary to hypothalamic activation (102).

## Thyroid hormones metabolites and the skeletal muscle

The skeletal muscle represents approximately 40% of body mass and significantly contributes to energy expenditure and oxygen consumption. Besides that, skeletal muscles contribute to the maintenance of blood glucose levels. Hence, skeletal muscles are important targets for the treatment of obesity (103).

Thyroid hormones influence both the type and distribution of the skeletal muscle fibers, their metabolic program and contractility apparatus (104). Muscular disorders occur in both hypo- and hyperthyroid patients (105). In skeletal muscle, T3 increases the transcription of MyoD (106), stimulates myosin heavy chain IIa (MHC IIa) (107) and the sarcoendoplasmic reticulum adenosine triphosphatase 1 isoform (SERCA1) expressions (108) through genomic actions. Under the effect of T3, the skeletal muscle energy metabolism is switched into an oxidative mitochondrial metabolism mediated by PGC-1α (50) and after exercise muscle D2 activity increases, which might be involved in the local T3 production necessary for these metabolic changes (109). T3 genomic actions significantly differ in muscle fibers due to the pattern of TR expression that are more abundant in slow-twitch than in fast-twitch muscles (110). As well as T3, 3,5-T2 also caused a slow to fast-twitch muscle transition and induced a shift toward a glycolytic phenotype, increasing key enzymes of glycolysis, such as phosphofructokinase (PFK) (89).

Apart from the well-known genomic actions of T3 in skeletal muscle, T3 administration increases the phosphorylation of AMPK and P38, preferentially in slow-twitch when compared to fast-twitch muscles (60). Both genomic and non-genomic pathways have been described to control mitochondrial biogenesis, a crucial step by which thyroid hormones can increase oxygen consumption.

When hypothyroid rats receive a single dose of 3,5-T2, AMPK phosphorylation increases in gastrocnemius muscle, a type of muscle that presents both slow- and fast-twitch fibers, while a single injection of T3 also induced the phosphorylation of both AMPK and acetyl CoA carboxylase (ACC) and a persistent phosphorylation of Akt. These changes lead to increased carnitine palmitoyl transferase (CPT) activity, fatty acid oxidation and increased GLUT4 translocation to the membrane (59). In rats fed a HFD, 3,5-T2 long-term administration enhanced Akt phosphorylation in skeletal muscle, increased lipid oxidation and ameliorated insulin resistance (38).

AMPK activation is a potential candidate to mediate the increased fatty acid oxidation detected in skeletal muscle after TH administration. AMPK activity was augmented by T3 in euthyroid (60) and hypothyroid rats (59) and by 3,5-T2 in euthyroid HFD fed (62) and hypothyroid rats (111). AMPK phosphorylation increased in the gastrocnemius of hypothyroid rats 1 hour after 3,5-T2 administration, which might explain the increased fatty acid oxidation (111).

T3 stimulates the electron transfer from cytosolic NADH through the mitochondrial alpha-glycerophosphate dehydrogenase (α-GPD), promoting a consequent loss of chemical energy as heat, due to the synthesis of only two ATP molecules from NADH instead of three (112). Differently, the acute treatment of hypothyroid rats with 3,5-T2 was sufficient to increase oxygen consumption related to FAD metabolism and did not affect the mitochondrial respiration when it was stimulated with malate, suggesting that the 3,5-T2 effect is independent of NADH metabolism and supporting the idea that lipid beta oxidation is responsible for the increased oxygen consumption (111).

The alteration in skeletal muscle mitochondrial proton conductance could also affect energy expenditure. A single dose of 3,5-T2 on mitochondrial parameters of hypothyroid rats was able to induce mitochondrial uncoupling due to increased substrate oxidation and the proton leak, probably through non-genomic actions (111). Both T3 and 3,5-T2 treatment for 4 weeks increased the UCP3 mRNA expression levels in skeletal muscle in diet induced obesity in mice (54).

Lastly, another effect of T3 is the ability to increase glucose uptake independent of insulin in skeletal muscle cells. T3 rapidly increases GLUT4 expression in skeletal muscle and its trafficking to the plasma membrane (113). In L6 muscle cells an increased glucose uptake independent of GLUT was reported in the first 30 min of T3 action (114). 3,5-T2 was also able to up-regulate sarcolemma membrane-associated GLUT4 protein content followed by increased insulin sensitivity in HFD fed rats (89). In contrast, another study did not show any effect of 3,5-T2 in GLUT4 expression in skeletal muscle (54).

Apparently, T1AM has an opposite effect when compared with 3,5-T2 and T3 actions in skeletal muscle. T1AM administration increases lipolysis (44, 45) and decreases carbohydrate utilization, as demonstrated after respiratory quotient (RQ) analysis (44). Protein breakdown also increases in mice after administration of T1AM for 8 days, as observed by Nuclear Magnetic Resonance spectroscopy (45). Recently, T1AM decreased oxygen consumption and cell diameter in cultured C2C12 myotubes. Suppression of AKT phosphorylation and mTOR activation together with increased catabolic pathways, such as ubiquitin E3 ligase, were demonstrated after T1AM treatment (115).

## Thyroid hormones metabolites and the liver

Liver insulin resistance secondary to lipid accumulation is one of the initiating steps related to obesity induced by high fat diet (116). The mechanisms involved in the development of non-alcoholic fatty liver disease (NAFLD) are: (1) increased hepatic lipogenesis, and/or (2) higher lipolysis in adipocytes.

T3 regulates lipid metabolism in the liver and induces both fatty acid oxidation and lipogenesis. Apart from its ability to induce lipolysis, T3 also increases the expression of genes involved in hepatic lipogenesis, such as SPOT14, ACC, and fatty acid synthase (FAS) (117), and is responsible for increased plasma triglycerides and decreased serum cholesterol in humans (118).

T3 administration was not efficient to treat NAFLD, contrary to what was observed when rats received the selective TR beta agonist MB07811 that significantly increased hepatic fatty acid oxidation, and decreased liver steatosis (119). In relation to cholesterol, TR beta ligands act through increased expression of scavenger receptor B type I (SR-BI) that promotes hepatic uptake of cholesterol, lowering serum cholesterol levels (120). TR beta is probably the isoform that mediates most of the liver T3 effects on lipid metabolism (35). However, using a luciferase expression vector containing the human uncoupling protein 3 (UCP3) promoter, a known target of T3 through TR beta activation suggested that TR beta is not implicated in liver 3,5-T2 responses (62).

The regulation of the D1 gene by T3 is mediated by TR beta, and T3 stimulates both hepatic and kidney D1 activities (1) through genomic actions. Rats treated with 3,5-T2 have low serum T3 and increased hepatic and kidney D1 activities (39), suggesting a T3-like genomic effect of 3,5-T2 mediated by TR beta in the liver D1 regulation (rather than a non-genomic action). However, a recent study using rats fed with HFD showed that the expression levels of D1 mRNA increased with T3 but not with 3,5-T2 (116). However, the differences may be attributed to the different doses of 3,5-T2 used, the period of time of treatment and the animal model.

3,5-T2 was able to increase oxygen consumption of hepatocytes faster than T4 or T3 (36). Interestingly, it was demonstrated that T4 and T3 actions were dependent on deiodination since PTU pre-treatment attenuated their effects, suggesting that 3,5-T2 was the metabolite responsible for the effects of thyroid hormones on liver oxygen consumption (25). Due to its remarkable ability to increase hepatocytes oxygen consumption and the subsequent findings that it had minor board of genomic effects, 3,5-T2 was tested as a therapeutic agent to treat NAFLD in animal models [for review see (121, 122)]. 3,5-T2 treatment was shown to prevent or treat hepatic steatosis and obesity induced by HFD, increasing insulin sensitivity in rats (38). Senese et al. (116) compared T3 and 3,5-T2 administration in a HFD induced NAFLD model, and the decrease in liver lipid accumulation mediated by 3,5-T2 was accompanied by a down regulation in the expression of lipogenic enzymes like SPOT14, ACC, and FAS, different from T3 effects (116). In contrast, in rat liver mitochondria T1AM decreases oxygen consumption and increases reactive oxygen species release through the inhibition of complex 3 activity (123). Also, T1AM increase the content of glutathione in liver cells and thus their antioxidant ability (70).

There are several significant differences between T3 and its metabolite 3,5-T2, and T1AM, concerning their molecular mechanisms of action (31, 121). T3 stimulates both hepatic lipogenesis and fatty acid oxidation, while 3,5-T2, and T1AM induce fatty acid oxidation and inhibit lipogenesis.

Daily 3,5-T2 administration for 4 months increased AMPK activity and could explain the increased lipid oxidation by hepatocytes (38), decreased liver lipid accumulation, and increased insulin sensitivity, which could prevent the accumulation of lipid in liver and also in skeletal muscle (62). Part of these beneficial effects of 3,5-T2 were not altered by the inhibition of AMPK by compound C. Thus, sirtuin might also participate in the pathway activated by 3,5-T2 (62). It is well-known that sirtuin activation normalizes the expression of gluconeogenic and lipogenic enzymes in liver, and *in vitro* experiments demonstrated that 3,5-T2, and stimulates while T3 decreases sirtuin activity (62). Recently, T1AM treatment of overweight mice led to increased net weight loss and decreased cholesterol levels. Part of these T1AM beneficial effects seem to be a consequence of increased Sirtuin 6 and decreased Sirtuin 4 expressions (31). Additionally, It has been previously shown that 3,5-T2 induces SIRT1-mediated deacetylation of the promoter of SREBP- 1c in liver, reducing its expression (62).

In liver, insulin-dependent Akt activation suppresses gluconeogenesis through the regulation of key enzymes, such as phosphoenolpyruvate carboxykinase (PEPCK) and glucose 6 phosphatase (G6Pase) (124). Recently, Da Silva Teixeira et al. (54) have shown that neither T3 nor 3,5-T2 trigger hepatic Akt phosphorylation, suggesting that these ligands do not alter hepatic insulin sensitivity through Akt activation (54). However, the gluconeogenesis was stimulated by T1AM in perfused rat liver and HepG2 cells, concomitant to the stimulation of fatty acid oxidation (29). Most studies with 3,5-T2 demonstrated amelioration in glucose tolerance, insulin sensitivity and liver steatosis (38, 39, 62). Therefore, both 3,5-T2, and T1AM could be tested as therapeutic agents against NAFLD.

T1AM ameliorates lipid profile in a model of polycystic ovary syndrome induced by glucocorticoids administration (70). On the other hand, T1AM might also induce insulin resistance due to decreased carbohydrate utilization by the cells (44, 45) and to decreased insulin secretion by beta-cells of the pancreas islets (125, 126). T1AM intraperitoneally resulted in decreased insulin secretion probably through the activation of alpha2A adrenergic receptors (Adra2a) in the beta cells of the pancreas, since the effect is abrogated in Adra2a knockout mice (126). Recently, it was shown that T1AM reduces the ATP turnover, which was implicated in the decrease of insulin secretion upon glucose stimulation (125). However, this acute *in vivo* effect of T1AM was not observed when a lower dose of T1AM was used and for a longer period of time (67). In contrast, 3,5-T2 stimulates insulin secretion upon glucose stimulation (127).

In short-term fasted male mice, the intracerebroventricular (icv) injection of T1AM (26) caused improved memory, hypophagia, and also peripheral effects such as reduced peripheral insulin sensitivity and higher plasma glucose levels (Figure 3), again highlighting the opposite effects of T1AM on glucose handling in relation to T3 and 3,5-T2.

## Thyroid hormones metabolites and the kidney

Thyroid hormones affect kidneys size, weight, and structure. Hypothyroidism decreases, whereas hyperthyroidism increases kidney weight (128). Thyroid hormones affect the kidneys through direct as well as indirect mechanisms since T3 influences systemic hemodynamic parameters and exerts important cardiovascular effects.

As described for patients with myocardial infarction, low serum T3 levels are associated with poor prognosis of patients with chronic kidney disease (129). Notably, renal function was improved by T3 treatment in patients with renal failure and severe hypothyroidism (130). Daily T3 administration for 4 weeks decreased urinary albumin excretion and attenuated the collagen accumulation and renal fibrosis in a model of diabetic nephropathy in mice. T3 decreased TGFβ expression and increased PI3K activity in kidney; the treatment with PI3K inhibitor abolished the beneficial effect of T3 in all parameters of renal function (131).

In an experimental model of diabetic nephropathy, 3,5-T2 treatment for 12 weeks also reduced fibrosis markers such as Fibronectin, Collagen IV and TGFβ expression ameliorating the renal function (88). Also, the restauration of sirtuin expression and activity was observed in diabetic rats treated with 3,5-T2, and the presence of sirtinol, an inhibitor of deacetylases, abolished the positive effect of 3,5-T2. It has been suggested that persistent JNK1 activation leads SIRT1 inhibition through increased protein degradation (88) and 3,5-T2 treatment attenuated JNK phosphorylation, what could be the mechanism of protection induced by 3,5-T2 that is apparently different from the pathways activated by T3 that are dependent on PI3K.

We cannot rule out that the anti-hyperglycaemic effect of both T3 and 3,5-T2 might also contribute to their protective effects on diabetic nephropathy models.

## Final remarks

Substantial data described in the last 30 years show a consistent action of metabolites of thyroid hormones in several cell types and pathophysiological conditions, opening new chapters about the broad spectrum of thyroid hormones action, and the synthesis and action of their metabolites (Table 1).

**Table 1:** Overview of 3,5-T2, and T1AM effects on Core temperature, Heart, Insulin sensibility: Liver-adipocyte-muscle and Pancreas.

**Core temperature**	**Dose /100g b.w**.	**Experiment**	**Effect(s)**
**3,5-T2**			
(51)	25 μg	HypoT rats	Maintenance of temperature in cold environement
(40)	25-250 μg	HFD, mice	Increased core temperature (with 250 μg/100g b.w.)
(54)	125-1250 μg	HFD, mice	Increased core temperature (with 1250 μg/100g b.w.)
**T1AM**			
(27)	5 mg	Mice	Transitory hypothermia
(44)	5 mg	Hamster and Mice	Transitory hypothermia
**Heart**	**Dose /100g b.w**.	**Experiment**	**Effect(s)**
**3,5-T2**			
(38)	25 μg	Euthyroid rats	Heart weight and HR unchanged
(39)	25-50-75 μg	Euthyroid rats	Increased H/b.w. (only 75 μg/100g b.w.). HR unchanged
(40)	25-250 μg	HFD, mice	Increased H/b.w. (only 250 ug/100g b.w.)
(54)	125-1250 μg	HFD, mice	Increased H/b.w. (with both doses used)
(92)	0,1-10 μM	Isolated rat heart	Increased glucose consumption (0,1-1 μM)
			Reduced contractile performance (10 μM)
**T1AM**			
(27)	5 mg /ED50 = 29 μM	Mice/Isolated rat heart	Bradycardia/reduced cardiac output *in vitro*
(30)	18-38 μM	Isolated rat heart	Reduced HR and contractility performance
(94)	0.125-12.5 μM	Isolated rat heart	Cardioprotection after ischemia insult
**Liver-Adipocyte-muscle**	**Dose /100g b.w**.	**Experiment**	**Effect(s)**
**3,5-T2**			
(36)	1 pM	Liver from HypoT rats	Increased oxygen consumption
(38)	25 μg	Euthyroid rats HFD	Increased FAO, hepatic steatosis atteunuated
(62)	25 μg	Euthyroid rats HFD	Increased FAO, increased insulin sensitivity
(39)	25-50-75 μg.	Euthyroid rats	Increased insulin sensitivity
(54)	125-1250 μg	HFD, mice	Reduced hepatic glucose output
(116)	25 μg	HFD, euthyroid rats	Increased lypolysis and decreased lipogenesis genes
**T1AM**			
(123)	10^−7^-10^5^ M	Liver from HypoT rats	Reduced oxygen consumption, increased H_2_O_2_ release
(45)	1 mg	Mice (overweight)	Increased FAO
(70)	2.5 mg	Mice (PCOS)	Antilipogenic and enhanced protection to oxidative stress
(31)	1-2.5 mg	Mice (overweight)	Shift of metabolism from carbohydrates to lipids
**Pancreas**	**Dose /100g b.w**.	**Experiment**	**Effect(s)**
**3,5-T2**			
(62)	25 μg	HFD, euthyroid rats	Blood Insulin levels unchanged
(39)	25-50-75 ug	Euthyroid rats	Blood Insulin levels unchanged
(127)	0.1 nM/l - 0.1 μM/L	Human islet and cells	Increased insulin secretion.
(54)	125-1250 μg	HFD, mice	Reduced insulin levels (with 1250 μg/100g b.w.)
**T1AM**			
(126)	50 mg/10 uM(*in vitro*)	Mice/isolated islets	Increased blood glucose/Reduced insulin secretion *in vitro*
(73)	13 μg	Mice (icv)	Hyperglicemia
(67)	0.5 mg	Mice	Basal fasting glucose and glucose tolerance unchanged
(125)	100 nM	Murine Beta-Cells	Reduced insulin secretion

*HypoT, Hypothyroid; HFD, High fat diet; H/b.w, Heart weight/body weight; HR, heart rate; Icv, intracerebroventricular; FAO, Fatty acid oxidation; PCOS, polycystic ovary syndrome*.

The pathway for 3,5-T2 formation is believed to be the outer ring deiodination of T3 probably mediated by D2 that catalyses the removal of the outer-ring iodine from thyroxine (T4). Conversion of T3 into 3,5-T2 requires outer-ring deiodination, which can be catalyzed by either D1 or D2.

Nuclear receptors have high affinity for T3. T3 to T2 conversion promotes specific binding of 3,5-T2 to different isoforms of TRs, but this metabolite also exerts potent non-genomic actions that control many cellular reactions.

3,5-T2 conversion to T1AM (Deamination and deiodination reactions) produces a hormone metabolite that does not seem to bind to TR and apparently exert opposite effects in relation to its precursors biological actions.

rT3 is the product of D3 deiodination that was described as an inactivating enzyme, however rT3 can also be a precursor of T1AM, a metabolite that counteracts many classical T3 actions, such as tachycardia and body temperature regulation.

In the future, a better understanding about the differences in the cellular pathways regulated by T3 and its metabolites is of great interest in order to possibly unravel novel therapeutic targets for the control of prevalent diseases.

## Author contributions

All authors listed have made a substantial, direct and intellectual contribution to the work, and approved it for publication.

### Conflict of interest statement

The authors declare that the research was conducted in the absence of any commercial or financial relationships that could be construed as a potential conflict of interest.
